# Western diet, obesity and bariatric surgery sequentially modulated anxiety, eating patterns and brain responses to sucrose in adult Yucatan minipigs

**DOI:** 10.1038/s41598-020-76910-9

**Published:** 2020-11-18

**Authors:** Yentl Gautier, Damien Bergeat, Yann Serrand, Noémie Réthoré, Mathilde Mahérault, Charles-Henri Malbert, Paul Meurice, Nicolas Coquery, Romain Moirand, David Val-Laillet

**Affiliations:** 1 INRAE, INSERM, Univ Rennes, CHU Rennes, Nutrition Metabolisms Cancer, UMR1341, 35590 NuMeCan, Rennes, St Gilles France; 2 CHU Rennes, Service de Chirurgie Hépatobiliaire Et Digestive, Rennes, France; 3 INRAE, US1395, AniScan, St Gilles France; 4 CHU Rennes, Service Des Maladies du Foie Et Addictologie, Rennes, France

**Keywords:** Neuroscience, Psychology, Diseases

## Abstract

Palatable sweet/fatty foods overconsumption is a major risk factor for obesity and eating disorders, also having an impact on neuro-behavioural hedonic and cognitive components comparable to what is described for substance abuse. We hypothesized that Yucatan minipigs would show hedonic, cognitive, and affective neuro-behavioral shifts when subjected to western diet (WD) exposure without weight gain, after the onset of obesity, and finally after weight loss induced by caloric restriction with (RYGB) or without (Sham) gastric bypass. Eating behavior, cognitive and affective abilities were assessed with a spatial discrimination task (holeboard test) and two-choice feed tests. Brain responses to oral sucrose were mapped using 18F-FDG positron emission tomography. WD exposure impaired working memory and led to an “addiction-type” neuronal pattern involving hippocampal and cortical brain areas. Obesity induced anxiety-like behavior, loss of motivation, and snacking-type eating behavior. Weight loss interventions normalized the motivational and affective states but not eating behavior patterns. Brain glucose metabolism increased in gustatory (insula) and executive control (aPFC) areas after weight loss, but RYGB showed higher responses in inhibition-related areas (dorsal striatum). These results showed that diet quality, weight loss, and the type of weight loss intervention differently impacted brain responses to sucrose in the Yucatan minipig model.

## Introduction

The plethoric availability of palatable and energy-dense foods is considered a major factor in the emergence of eating disorders and obesity. Repeated exposure to high-energy food, notably during sensitive periods, is associated with hyperphagia and food cravings phenomena toward these foods, with the possibility of bypassing homeostatic regulation mechanisms^1^. Numerous works have described the parallel between the Western-type fatty/sweet foods overintake (chronic consumption with or without obesity) and drugs of abuse consumption. They have comparable effects on the neural circuitry of pleasure and cognition as well as on behavioral outcomes, such as eating behaviors, mood and anxiety- or depression-like symptoms, learning/memory, and general health^2–5^. Moreover, behavioral and cerebral features related to hedonic functions (including the dopaminergic system) are different in obese or overweight subjects compared to healthy human subjects^6–8^, as well as in rat and minipig obesity models^9,10^.

Interestingly, the most effective anti-obesity therapy to date is the bariatric surgery intervention, such as Roux-en-Y gastric bypass (RYGB). The RYGB drastically modifies eating behavior and leads to a generally rapid weight loss compared with a caloric restriction (dieting). It also induces profound brain anatomical and functional changes^11–13^, which could be a reverse switch compared to that observed during weight gain and the introduction of habitual consumption of palatable foods. Indeed, Le’s team^14^ had previously demonstrated that a successulf weigh-loss diet restored a normal activity in the prefrontal cortex in formerly obese women. But beyond its obvious therapeutic dimension, bypass surgery can be used as an experimental model to understand how the drastic modulation of the reward circuit might correspond to a reversed neuroplasticity phenomenon compared to that observed in the usual consumption of palatable foods and pathological weight gain. Also, the high invasiveness of bariatric surgery and the lack of efficiency of caloric restriction^15–17^ justify the development of research toward alternative therapies. Integrative and longitudinal preclinical studies in a relevant animal model such as the minipig might open the way to new developments in neurocognitive treatments.

Amongst the main questions that such a research thematic raises, is to what extent the chronic consumption of these palatable foods (independently of pathological weight gain) during the adolescent sensitive period can condition the alteration of neuronal plasticity and the establishment of a shift in the hedonic and cognitive processes, which regulate food choices and intake. We also do not know the respective roles of eating type (healthy or Western-type) and weight gain in this neuro-behavioral plasticity. Here, we present the first complete longitudinal study in adolescent minipigs assessing whether neuro-behavioral responses are impacted by i) chronic consumption of Western-type food without any weight gain (Study 1), ii) weight gain until obesity (Study 2), and iii) weight loss induced by a Roux-en-Y by-pass or restrictive diet (Study 3).

We hypothesized that chronic exposure to palatable foods in adolescent minipigs, independently of weight gain, should induce changes in perception, memory, and motivation for fatty and especially sweet stimuli, as well as changes in brain responses to food stimuli (*e.g.* in the hippocampus, striatum, and prefrontal cortex). Exposure to Western-type food should therefore induce i) a hedonic and motivational neuronal shift preceding the emergence of overweight, and ii) the modulation of cognitive processes in the longer term, with the emergence of obesity that may favor the onset of behavioral disorders (addiction, depression and/or anxiety-like behavior, which are closely related to anomalies of the reward and motivation circuit). By reshaping brain metabolism through interventional strategies (restrictive diet or obesity surgery) in the minipig, motivational and cerebral responses to sweet stimuli should be modified concomittantly, as well as some neurocognitive processes. The ultimate goal would be to restore the situation preceding the shift in neurocognitive and behavioural responses, and to clarify the role of brain structures that might be further targeted for selective neurocognitive treatments in the human, in addition or replacement to obesity surgery.

## Results

### Food intake, weight and survival rate overview (all studies)

#### Study 1

Animals were provided with an isocaloric amount of a standard diet (SD) or a high-fat-sucrose Western-type diet (WD) during 8 weeks prior to start the tests, and continued with their respective diets throughout the testing period (about 2 months). Both lean SD (N = 8) and lean WD (N = 20) received a feed ration corresponding to 4.8 ± 0.2 MJ/day (SD feed: 650 and 680 g/day for females and males respectively; WD feed: 450 and 480 g/day for females and males respectively). Lean SD and WD had the same live body weight (*P* = 0.69).

#### Study 2

**Figure 1:**
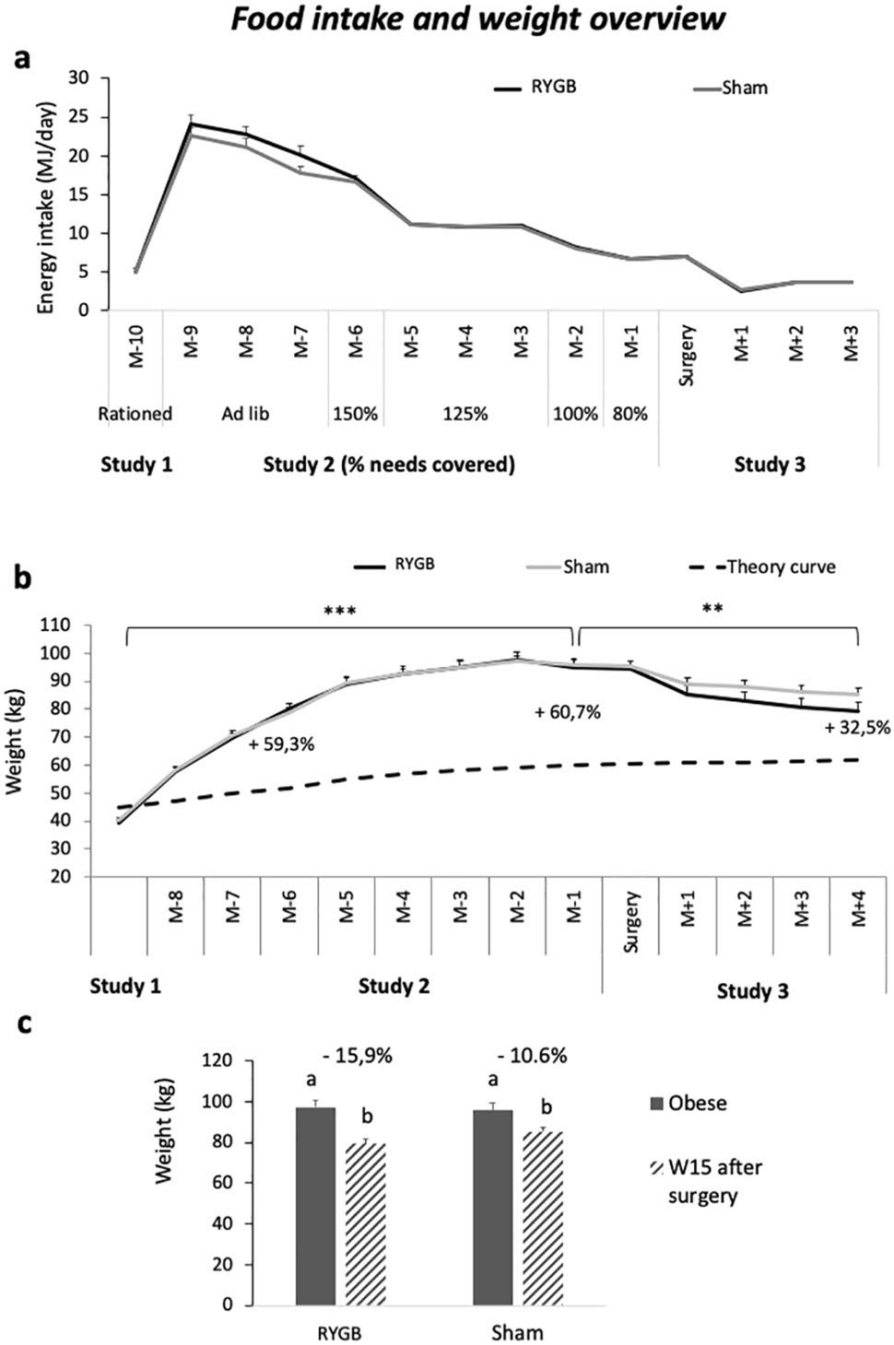
*Food intake and weight overview*. (**a**) Daily food intake (MJ) during Study 1 (Lean), Study 2 (Obesogenic challenge), and Study 3 (Intervention). Time is presented as months before (M-) and after (M +) surgery. During the Ad libitum phase, animals spontaneously reduced their food consumption, resulting in a significant decreased of daily energy intake. The percentage of feed ration indicated in the figure corresponds to energy needs covered according to M-6 animals’ body weight used as reference (when animals just reached the morbidly obese status) to calculate energy restriction until surgery. (**b**) Animals’ body weight (kg) compared to the Yucatan minipigs’ theoretical healthy weight curve (black doted line), according to the time (months), in both RYGB (black full line) and Sham (grey full line) groups. Percentages of excess weight are presented (calculated according to the theoric normal weight at the same age), with *P < 0.05, **P < 0.01, ***P < 0.001. (**c**) Body weight (kg) in obese (full grey), and 15 weeks after intervention (hatched grey) in both RYGB and Sham-operated animals, with respective percentage of weight loss.

When fed ad libitum with high-fat-sucrose feed, WD animals from Study 1 (N = 20) increased their feed intake to reach a maximum average of 23.4 ± 0.4 MJ/day the first month of obesogenic challenge (nine months before surgery, M-9). Before the new rationing (intented to stabilize animals’ weight from M-6), the animals spontaneously reduced their food intake between the first and the fourth month of ad libitum diet (*i.e.* from M-9 to M-6 before surgery), presenting a significant decrease from M-9 (t = 2.27 to 8.97, df = 16, *P* < 0.008 Bonferroni corrected threshold in all cases). Animals were gradually rationed from the fourth month of obesogenic diet (M-6) until surgery (Fig. 1a).

Compared to initial healthy weight in Study 1, obese WD animals in Study 2 gained a maximum average 145% weight during the obesogenic phase between 12 and 24 months of age, and were significantly heavier than when they were lean WD in Study 1 (*P* < 0.001, Fig. 1b). They reached a weight 60.7% higher than the theoric growth curve at 2 years of age (24 months), defining them as morbidly obese.

The tests started after 4 months of obesogenic challenge (between M-5 & M-4), the testing period lasted about 3 months (ended between M-2 & M-1).

#### Study 3

After the weight loss intervention (bypass surgery) performed on obese WD animals from Study 2, animals (RYGB, N = 8) presented an average 1.6 ± 0.9 days of anorexia, and they took 11.7 ± 0.8 days in average to reach a normal 500-g amont of feed consumed. One animal was excluded from these data because it presented an atypical behavior, with 13 days of anorexia, and took 24 days to reach the normal expected 500-g feed consumption per day. Fifteen days after surgery, 87.5% of animals succeeded in eating the complete 500-g amount of feed, and 100% of animals reached it 24 days after surgery. Because one animal died 2 months after surgery, another one (from the 4 spare animals) was operated to replace him. The survival rate was of 88.8% (8/9 animals having undergone the gastric bypass survived).

The animals from the RYGB group were compared to the sham-operated animals (Sham, N = 8, also from the obese WD animals from Study 2) were less impacted by surgery as expected. Three animals among the eight ate lower than the 100-g feed ration allocated the first day post-surgery, and only one on the second day. From the third day after surgery, all animals were able to consume the entire allocated ration, in pair-feeding with RYGB animals. One month after surgery, both RYGB and sham-operated animals received 500 g of standard feed per day providing 3.6 MJ/day.

Four months (15 weeks after surgery, W15) after intervention, animals’ weight was lower than when they were obese (between 24 and 28 months of age, *P* < 0.01), in both RYGB (t = 30.4, df = 6, *P* < 0.001) and Sham (t = 12.2, df = 7, *P* < 0.001) groups. The body weight of RYGB and Sham animals did not differ significantly (79.3 ± 3.5 kg and 85.2 ± 2.4 kg, respectively, *i.e.* 15.9 ± 0.5% and 10.6 ± 1.0% of weight loss compared to P2; *P* = 0.35, Fig. 1c). Fifteen weeks after surgery (28 months of age), animals presented 32.5% of excess weight compared with the theoretical growth curve, defining them as moderately obese.

No sex effect was found, neither in lean SD and WD (sex: diet, F_1,24_ = 0.29, *P* = 0.59), Obese (sex effect, F_1,15_ = 0.009, *P* = 0.92), nor after intervention for RYGB and Sham (sex:status, F_1,12_ = 0.36, *P* = 0.56).

The tests started 2 months after interventions and lasted about 2 months.

### Study 1: Isocaloric western diet compared to standard diet impaired memory and induced “addiction-like” brain activation patterns in normal weight animals (lean SD vs. WD)

#### Holeboard results

**Figure 2:**
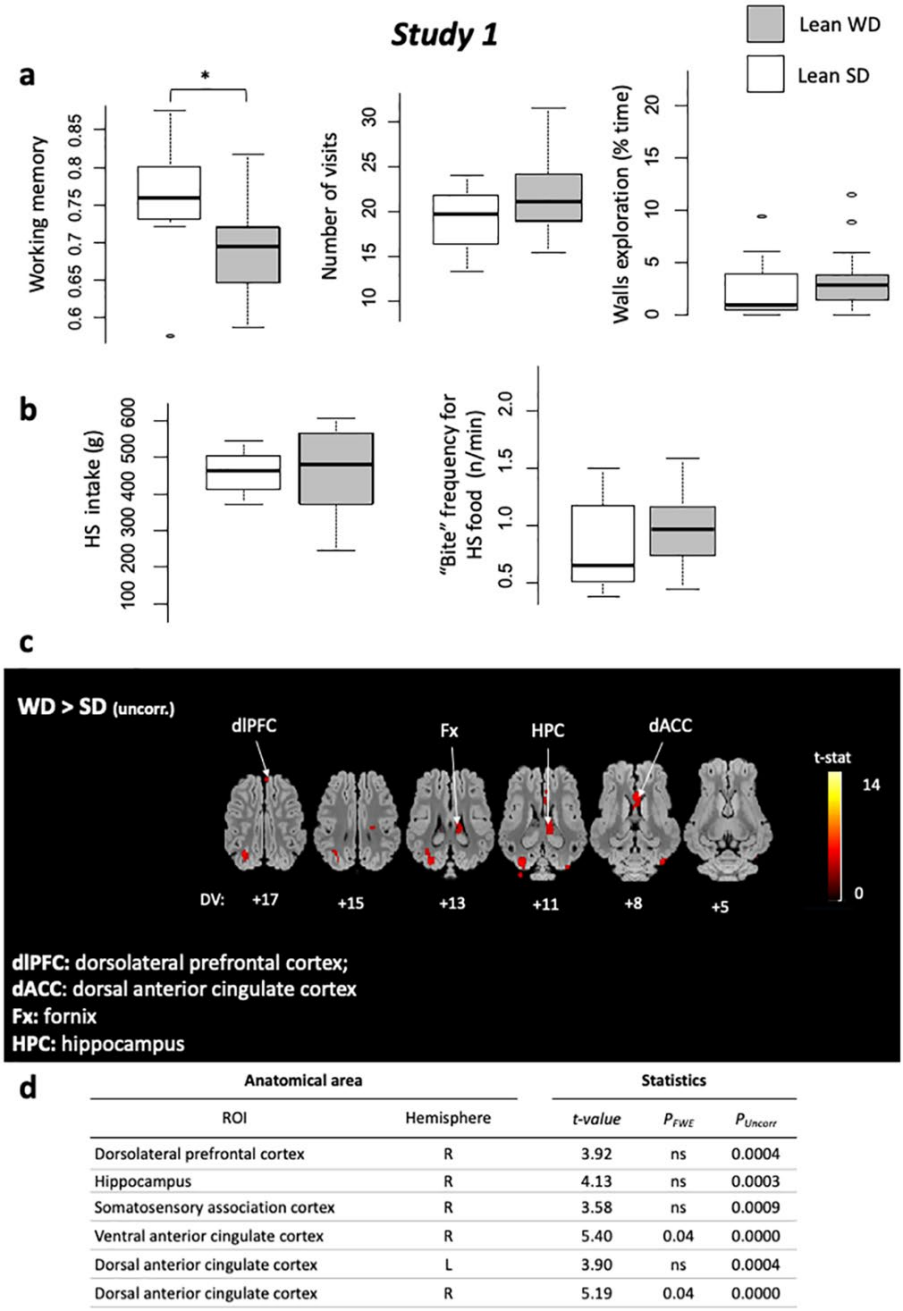
*Study 1*. **(a)** Holebard discrimination task results. Comparisons between lean SD (white) and lean WD (light grey) for Working memory scores, Number of bowl visits (occurences), and Walls exploration time (s). With *P < 0.05, **P < 0.01, ***P < 0.001. (**b)** Two-choice feed test results. Comparisons between lean SD (white) and lean WD (light grey), for High-sucrose feed intake (g) and “Bite” frequency for HS food (occurences/min). (**c)** Horizontal brain maps (whole-brain analyses) of higher glucose metabolism in response to sucrose in WD compared to SD lean animals (*P* < 0.001, uncorrected); DV, dorsal–ventral position in mm related to the posterior commissure. **(d)** Brain areas for which higher glucose metabolism was detected after a ROI (regions of interest) analysis based on a priori hypotheses. The contrasts and statistical thresholds (at the peak) are the same as those presented for the whole-brain analysis.

No difference appeared between lean SD and WD for the reference memory (RM, *P* = 0.20), but SD showed higher working memory (WM) scores than WD during the acquisition phase (*P* < 0.05), associated with higher test duration (*P* < 0.05) than SD. No difference arose between groups in terms of motivation or anxiety-like behaviors according to immobility (*P* = 0.78), exploration of the walls (*P* = 0.59), vigilences (*P* = 0.25), and vocalizations (*P* = 0.87; Fig. 2a).

#### Eating behavior (two-choice test)

The high-sucrose feed (HS) was largely prefered over the low-sucrose feed (LS) used in the two-choice feed test in all studies (1: Lean, 2: Obese, and 3: Post-intervention), in both lean SD and WD groups (HS = 433.5 ± 28.4 g, LS = 108.3 ± 16.8 g, V = 230, *P* < 0.001). The quantity ingested was similar between SD and WD groups for HS feed (*P* = 0.57). Both groups showed a similar meal microstructure in terms of HS ingestion rate (*P* = 0.14), “bite” (*P* = 0.32; Fig. 2b) and “sniff” frequencies (*P* = 0.23).

#### Brain glucose metabolism changes in response to sweet taste (PET imaging)

##### Whole-brain analysis

Sweet taste stimulation produced peaks of activation in the fornix (Fx) and the dorso-lateral prefrontal cortex (dlPFC) in WD compared to SD. The hippocampus (Hpc) and the dorsal-anterior cingulated cortex (dACC) were also covered by activated clusters, Fig. 2c,d).

### Study 2: Obesity induced anxiety-like behavior, decreased motivation, and triggered a snacking-type eating behavior (Lean WD vs. Obese WD)

#### Food preferences (two-choice test)

The HS feed was largely prefered over the LS feed in obese animals (HS = 362.5 ± 31.1 g, LS = 124.3 ± 18.0 g, V = 120, *P* < 0.001), after intervention in RYGB (V = 21, *P* < 0.05), and in sham-operated animals (V = 36, *P* < 0.01). As a consequence, the following results for the two-choice test, including Study 3, will concern only behavior toward the high-preferred HS food.

#### Obesity effects on behavior

##### Memory (holeboard)

**Figure 3:**
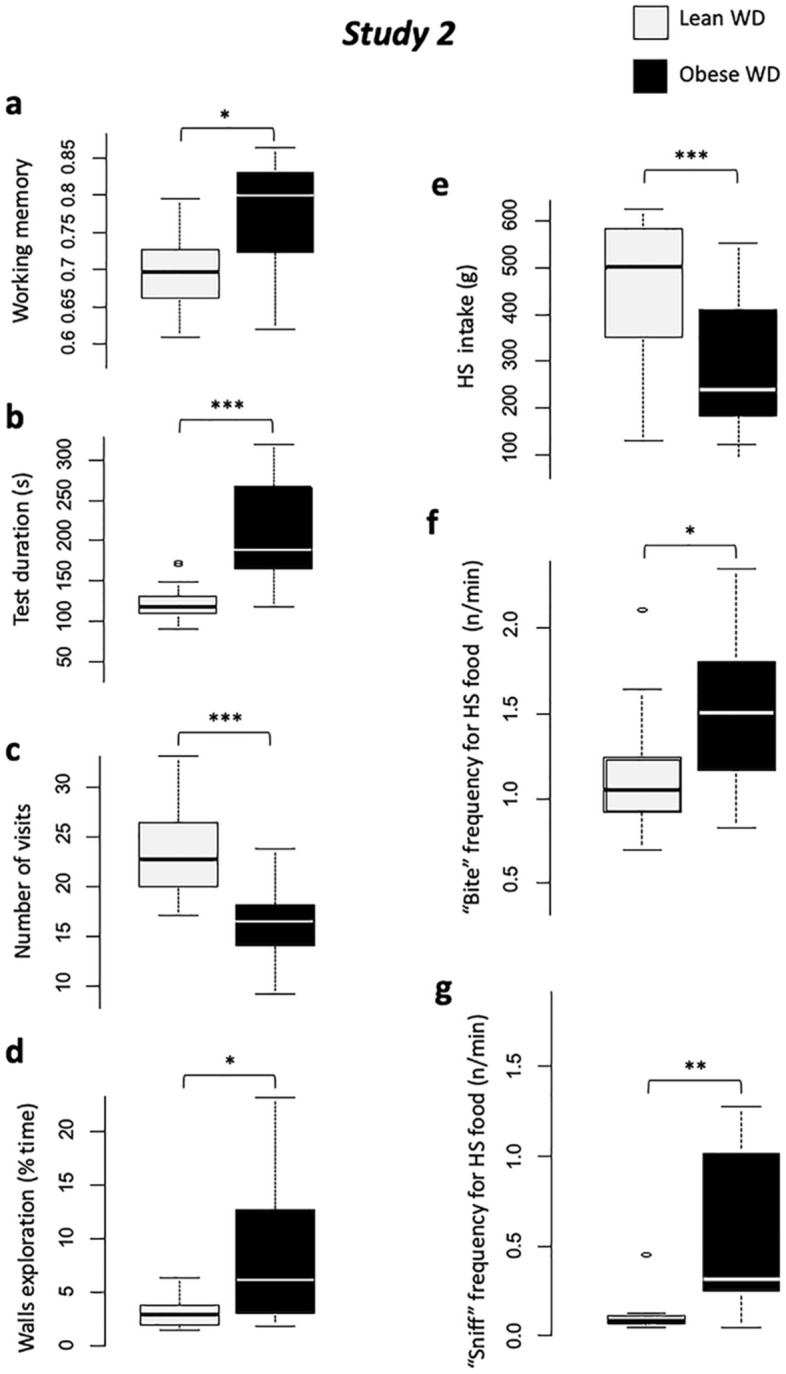
*Study 2. *Comparisons between lean WD (light grey) and obese (black), for holebard discrimination task results: (**a**) Working memory scores, (**b**) Test duration (s), (**c)** Number of bowl visits (occurences), and (**d**) Walls exploration time (s); and two-choice feed test: (**e**) High-sucrose feed intake (g), (**f**) “Bite” behavior frequency (occurences/min), (**g**) “Sniff” behavior frequency (occurences/min). With #*P* < 0.10, *P < 0.05, **P < 0.01, ***P < 0.001.

Scores were a little higher in obese animals compared to lean WD animals for WM (*P* < 0.05, Fig. 3a) and RM (V = 29, *P* < 0.01), but this result has to be considered under the light of exploration (*i.e.* bowl visits) results that strongly decreased in obese animals (see below), and in consequence, decreased the error probability.

##### Motivation (holeboard)

Test duration was significantly higher in obese than lean animals (*P* < 0.001, Fig. 3b). Obese animals showed higher first visit latency (*P* < 0.01), higher eaten/available reward ratio (*P* < 0.01), lower number of visits (*P* < 0.001, Fig. 3c), and higher switch time (going from one bowl to another (*P* < 0.001) than lean animals. These results show that obesity negatively impacted the animals’ motivation for the test.

##### Attentional/emotionnal status (holeboard)

Obese animals showed lower vigilance (*P* < 0.05), spent more time exploring walls (*P* < 0.05, Fig. 3d), and produced more vocalizations (*P* < 0.01), which illustrates a higher social contact seeking and anxiety-like behaviors in obese animals.

##### Eating behavior (two-choice test)

When obese, animals ate less HS feed than when they were lean (*P* < 0.001, Fig. 3e). Quantity ingested was not influenced by interaction between status and sex. The HS feed ingestion rate was also higher when animals were lean compared to obese (*P* < 0.01). Meal microstructure was altered by obesity development: *“*bite” (Fig. 3f) and “sniff” (Fig. 3g) frequencies significantly increased with obesity compared to lean status (*P* < 0.05 and *P* < 0.01 for “eat” and “smell” frequency respectively), representing a snacking-type eating behavior.

#### Brain glucose metabolism changes in response to sweet taste (PET imaging)

Comparison between lean and obese status is not available because of experimental constraint described in the M&M (position of animals differed during imaging: prone in Study 1, supine in Study 2).

### Study 3: Weight loss restored motivational state, reduced anxiety, and enhanced brain activity in sensory processes areas. Eating behavior modulation was intervention-type-dependant (Obese vs. Intervention and RYGB vs. Sham operated)

#### Weight loss effects on behavior (RYGB and Sham pooled together)

##### Memory (holeboard)

**Figure 4:**
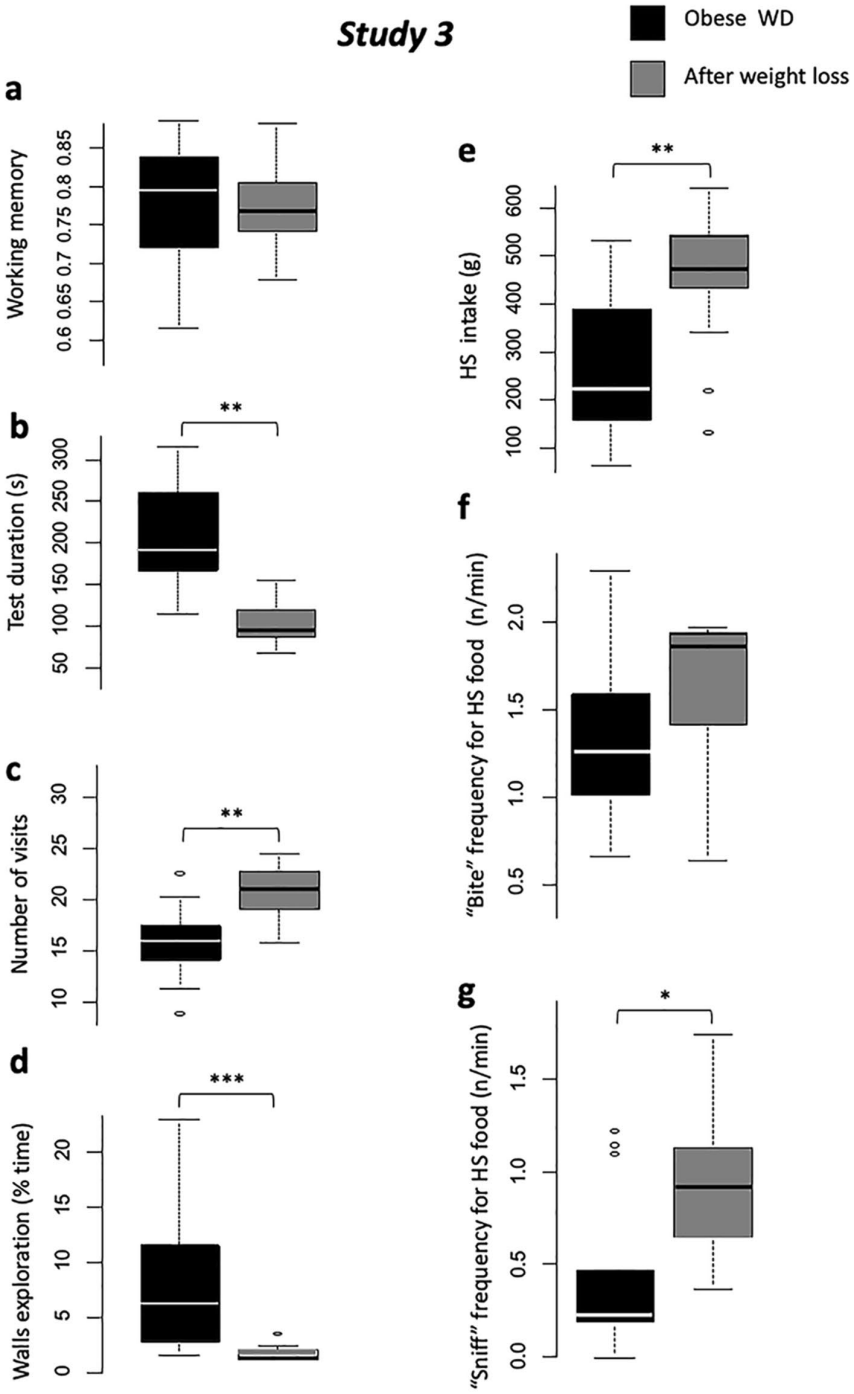
*Study 3. *Comparisons between obese (black) and post-interventions with RYGB and Sham pooled together (dark grey), for holebard discrimination task results: (**a**) Working memory scores, (**b**) Test duration (s), (**c**) Number of bowl visits (occurences), and (**d**) Walls exploration time (s); and two-choice feed test: (**e**) High-sucrose feed intake (g), (**f**) “Bite” behavior frequency (occurences/min), (**g**) “Sniff” behavior frequency (occurences/min). With #*P* < 0.10, *P < 0.05, **P < 0.01, ***P < 0.001.

No difference arose between obese and post-intervention status (RYGB and Sham), neither for WM (*P* = 0.79, Fig. 4a) nor RM (*P* = 0.34).

##### Motivation (holeboard)

Test duration was significantly lower post-intervention compared to the obese phase (*P* < 0.01, Fig. 4b). The first visit latency and switch time were lower post-intervention (*P* < 0.001 for both), and animals ate more rewards (*P* < 0.01) and visited more bowls (*P* < 0.01, (Fig. 4c) compared to the obese phase showing a motivation recovery after weight loss.

##### Attentional/emotionnal status (holeboard)

Animals showed less anxiety-like behavior after weight loss: spent less time immobile (*P* < 0.05) and exploring the walls (*P* < 0.001, Fig. 4d), and expressed less vocalizations (*P* < 0.001) in the post-intervention compared to obese phase.

##### Eating behavior (two-choice test)

Animals ate more HS feed after intervention than when they were obese (*P* < 0.001, Fig. 4e), with a higher ingestion rate (*P* < 0.01). Although meal microstructure was not impacted in terms of “bite” frequency (*P* = 0.11, Fig. 4f), weight loss increased “sniff” frequency compared to obese (*P* < 0.05, Fig. 4g).

#### Intervention type effects on behavior (RYGB vs. Sham)

##### Memory (holeboard)

**Figure 5:**
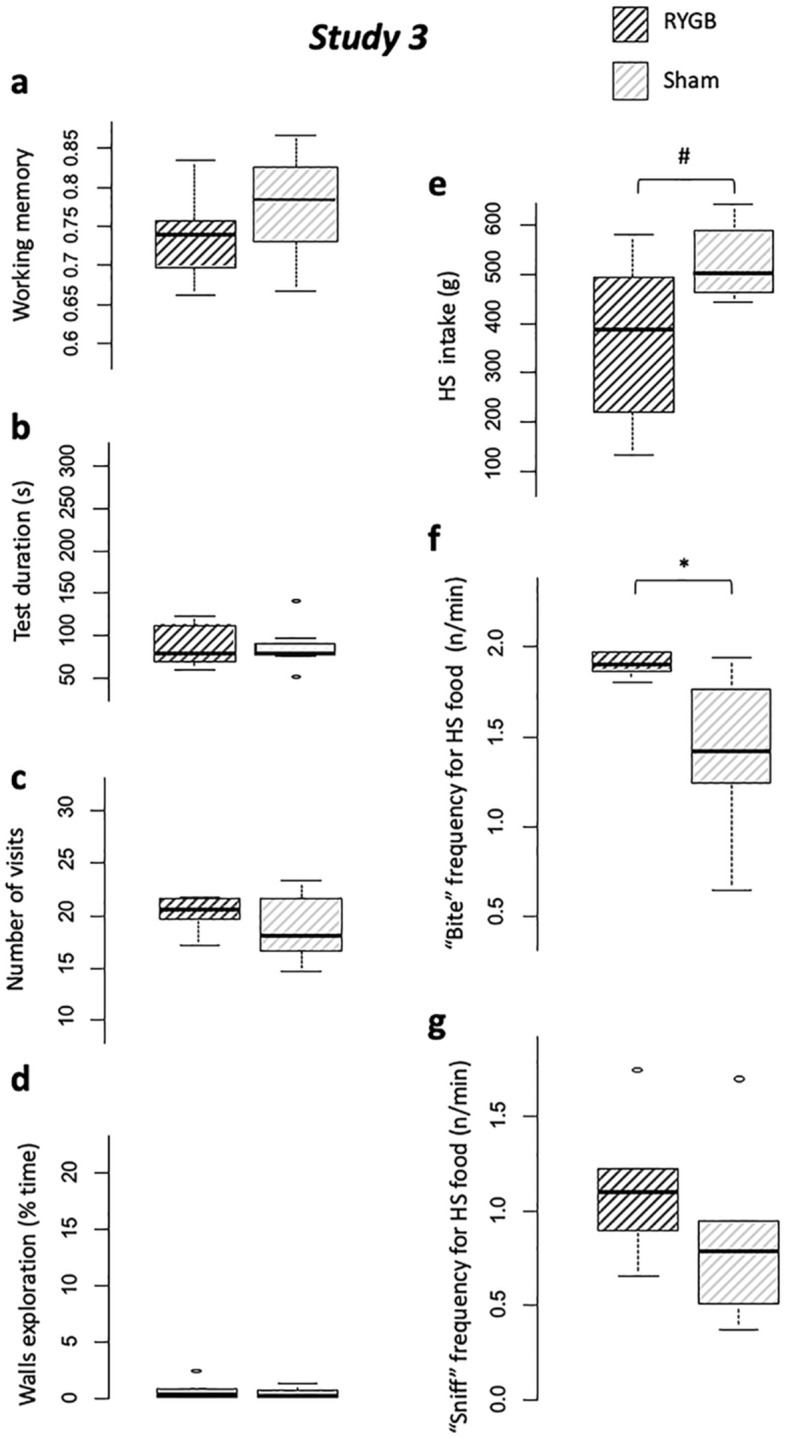
*Study 3.* Comparisons between between RYGB (hatched dark grey) and Sham (hatched light grey), for holeboard discrimination task results: (**a**) Working memory scores, (**b**) Test duration (s), (**c**) Number of bowl visits (occurences), and (**d**) Walls exploration time (s); and two-choice feed test: (**e**) High-sucrose feed intake (g), (**f**) “Bite” behavior frequency (occurences/min), (**g**) “Sniff” behavior frequency (occurences/min). With #*P* < 0.10, *P < 0.05, **P < 0.01, ***P < 0.001.

No difference appeared between RYGB and Sham in terms of memory scores, neither for WM (*P* = 0.18, Fig. 5a) nor RM (*P* = 0.37).

##### Motivation (holeboard)

The RYGB and Sham did not differ in terms of test duration (*P* = 1.0, Fig. 5b). Switch time (*P* = 0.53), eaten rewards ratio (*P* = 0.38), first visit latency (*P* = 0.10), and number of visits (*P* = 0.63, Fig. 5c) did not differ between RYGB and Sham groups.

##### Attentional/emotionnal status (holeboard)

The time spent exploring the walls (*P* = 0.74, Fig. 5d), the number of vocalizations (*P* = 1), the number of vigilences (*P* = 0.79) and immobilities (*P* = 0.31) did not differ between RYGB and Sham groups.

##### Eating behavior (two-choice test)

Quantity ingested tended to be lower in RYGB than Sham (*P* = 0.08, Fig. 5e), but ingestion rate was not different between groups (*P* = 0.10). RYGB showed a trend toward higher “bite” frequency (*P* = 0.05, Fig. 5f) compared to Sham, but no difference appeared for “sniff” frequency (*P* = 0.14, Fig. 5g).

#### Brain glucose metabolism changes in response to sweet taste (PET imaging)

##### Whole-brain analysis

**Figure 6:**
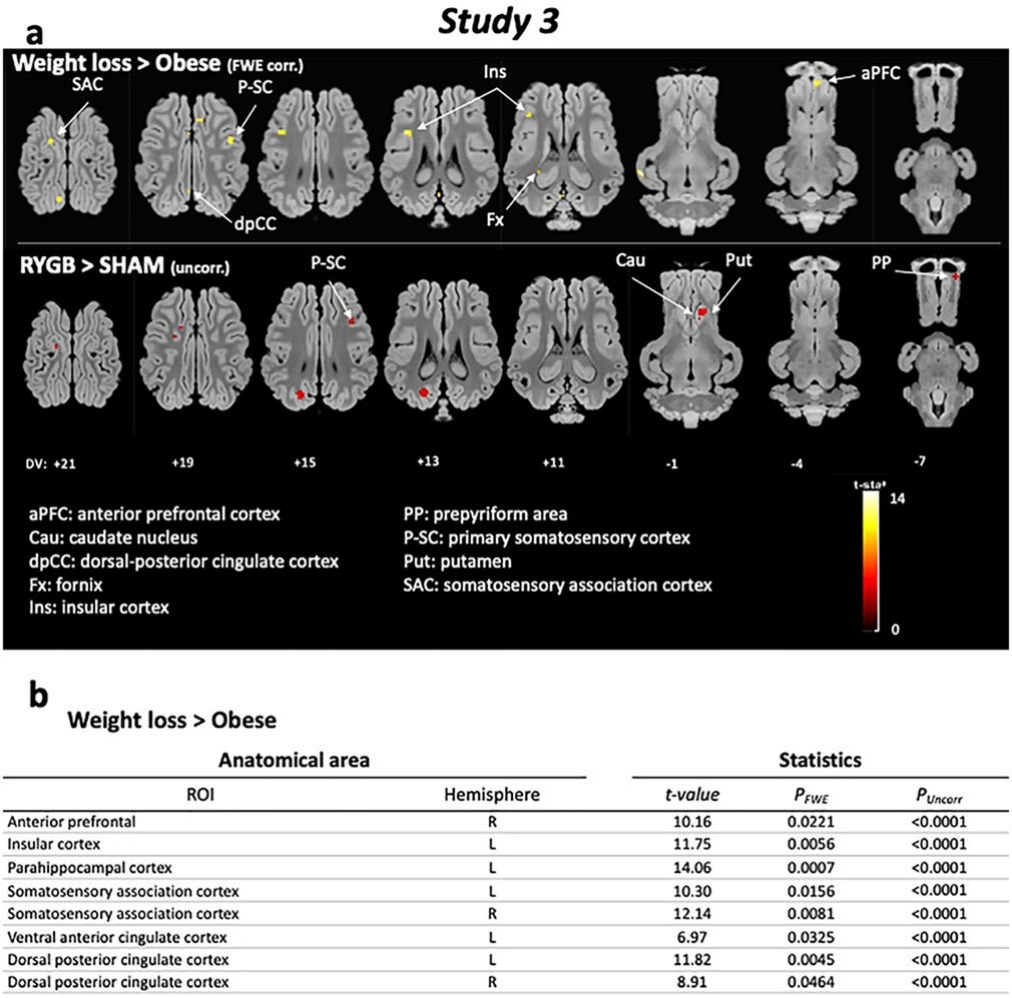
*Study 3*. **(a)** Horizontal brain maps (whole-brain analyses) of higher glucose metabolism in response to sucrose in post-interventions (RYGB and Sham pooled) compared to pre-surgery obese animals (P2) (*P* < 0.05 after FWE correction), and in RYGB compared to Sham animals (*P* < 0.001, uncorrected); DV, dorsal–ventral position in mm related to the posterior commissure. **(b)** Brain areas for which higher glucose metabolism was detected after a ROI (regions of interest) analysis based on a priori hypotheses (SVC approach). Only significant differences obtained after family-wise error (FWE) correction are indicated here. For the whole table including uncorrected significant differences, please see Table 1.

Compared to obesity, weight loss intervention induced higher metabolism in fornix (Fx), insula (Ins), dorsal-posterior cingulated (dpCC), primary somatosensory cortex (P-SC), somatosensory association cortex (SAC), and anterior prefrontal cortex (aPFC). The RYGB intervention involved peaks of activation in the P-SC, putamen (Put), caudate nucleus (Cau), and prepyriform area (PP) compared to Sham intervention (Fig. 6a).

##### ROIs analysis

The ROI-based analysis confirmed these results and also showed higher metabolism in Hpc, SAC, ventral anterior cingulated (vACC), and dACC in WD compared to SD (*P* < 0.001, uncorrected), in aPFC, Ins, parahippocampal cortex (PHC), SAC, and dACC after weight loss intervention compared to obese status (*P* < 0.05 after FWE correction), and in Cau, Put, SAC, and dpCC in RYGB compared to Sham group (*P* < 0.001, uncorrected) (Fig. 6b, Table 1).

**Table 1:** Brain areas for which higher glucose metabolism was detected after a ROI (regions of interest) analysis based on a priori hypotheses (SVC approach). The contrasts and statistical thresholds (at the peak) are the same as those presented for the whole-brain analysis presented in Fig. 6.

Anatomical area		Statistics		
ROI	Hemisphere	*t-value*	*P*_*FWE*_	*P*_*Uncorr*_
**Weight loss > Obese**				
Dorsolateral prefrontal	L	7.64	ns	< 0.0001
Dorsolateral prefrontal	R	6.19	ns	< 0.0001
Anterior prefrontal	L	7.39	ns	< 0.0001
Anterior prefrontal	R	10.16	0.0221	< 0.0001
Orbitofrontal cortex	L	4.51	ns	0.0004
Orbitofrontal cortex	R	5.39	ns	0.0001
Caudate nucleus	L	6.11	ns	< 0.0001
Caudate nucleus	R	8.63	ns	< 0.0001
Putamen	L	9.28	ns	< 0.0001
Putamen	R	8.36	ns	< 0.0001
Accumbens nucleus	L	5.78	ns	0.0001
Accumbens nucleus	R	5.57	ns	0.0001
Globus pallidus	L	6.63	ns	< 0.0001
Globus pallidus	R	6.31	ns	< 0.0001
Insular cortex	L	11.75	0.0056	< 0.0001
Insular cortex	R	7.69	ns	< 0.0001
Hippocampus	L	6.61	ns	< 0.0001
Hippocampus	R	6.26	ns	< 0.0001
Parahippocampal cortex	L	14.06	0.0007	< 0.0001
Parahippocampal cortex	R	7.77	ns	< 0.0001
Somatosensory association cortex	L	10.30	0.0156	< 0.0001
Somatosensory association cortex	R	12.14	0.0081	< 0.0001
Ventral posterior cingulate cortex	L	5.71	ns	0.0001
Ventral posterior cingulate cortex	R	4.65	ns	0.0004
Ventral anterior cingulate cortex	L	6.97	0.0325	< 0.0001
Ventral anterior cingulate cortex	R	7.11	ns	< 0.0001
Dorsal posterior cingulate cortex	L	11.82	0.0045	< 0.0001
Dorsal posterior cingulate cortex	R	8.91	0.0464	< 0.0001
Dorsal anterior cingulate cortex	L	7.10	ns	< 0.0001
Dorsal anterior cingulate cortex	R	7.02	ns	< 0.0001
**RYGB > Sham**				
Caudate nucleus	R	4.28	ns	0.0005
Putamen	R	4.81	ns	0.0002
Somatosensory association cortex	L	5.42	ns	0.0001
Dorsal posterior cingulate cortex	L	4.42	ns	0.0004

## Discussion

This work provides an integrative analysis of the respective effects of 1) diet quality, 2) weight gain, and 3) weight-loss interventional strategies on the behavioural/cognitive outcomes and brain responses to sweet taste. The discussion will be organized according to the three steps of our study.

*Study 1: Diet quality effect in lean individuals*. The isocaloric rationing allocated during the first phase of this project allowed both SD and WD animals to present a weight evolution matching with healthy theoretical growth curve. The lean SD and WD animals differed in terms of working memory during the holeboard discrimination task, where WD showed lower WM scores than SD. The WD animals also showed higher brain responses to sucrose stimulation in dlPFC, Hpc, and dACC.

According to the literature, high-fat-sugar diet associated or not with obesity has been identified as a factor able to impair memory and cognitive processes^3,18^. Both long-term exposure^19,20^ and short-term exposure^20–22^ may have deleterious effects on memory skills. It has been shown that short-term exposure to high-fat diet altered attention in the human^23^ resulting in WM impairment. In the present work, it is not possible to determine whether lower WM scores observed in WD animals was linked to long- (8-week exposure) and/or short-term exposure (meal before testing) to high-fat-sucrose diet, but our results are consistent with previous studies in this field. Some authors investigated glucose short-term effect on memory retention and showed a memory enhancement, but most of these studies were conducted among elderly and/or unhealthy humans^24–27^, or explored long-term effects of perinatal exposure to energy-dense diet^28,29^, which cannot be compared with the present work on young healthy animals.

Investigating brain responses to taste stimulation in Yucatan minipigs has already been validated as a relevant model for human, because similar brain networks are involved^30^. Brain responses to oral sucrose stimulation encompassed a higher glucose metabolism in dlPFC, Hpc and dACC in WD compared to SD, which could be interpreted in regards with current literature about food addiction and/or incentive sensitization. Humans subjected to food cravings present similar neuronal patterns to those involved in drug craving^31^, notably in the hippocampus, which contributes to support the concept of food addiction. Notably, human studies revealed a hyperactivation in dlPFC in bulimic and food addict people^32,33^ in response to food reward anticipation. A similar increase in dlPFC has been observed in obese children in response to food stimuli^34^. In rats, cocaine-seeking behavior has been triggered again by hippocampal electrical stimulation after the extinction of this behavior, revealing the major role of hippocampus in retrieval memory cues of stimulus-reward association and in relapse^5,35^. Taken alone, dlPFC is known to be involved in attentional processes and dorsal Anterior Cingulate Cortex (dACC) plays a major role in awareness^36^. In the present study, WD animals might have experienced a “sugar memory” during brain imaging with sucrose stimulation, in relation to the hippocampus activation. The activations in dlPFC and dACC might reveal high-focused attention processes toward the sweet taste, a neuronal pattern potentially reflecting addiction or at least habituation to sweet food. We must reckon though that the behavioral criteria for addiction were not met in this study. Our brain imaging results are consistent with the results showed by Winterdahl and collaborators^37^, who found that chronic exposure to sucrose reduced D2/D3 dopamine receptors and µ-opioid receptors availability in Göttingen minipigs’ prefrontal cortex, cingulated and striatum. The D2/D3 receptors support an inhibiting message, and a lack of these D2-like receptor result in altered inhibition in goal-directed behaviour. This could explain the higher metabolism in brain dlPFC, cingulate cortex and hippocampus showed in the present study.

### Study 2: Effect of obesity development

The WD animals were overly fed a high-fat-sucrose diet and became morbidly obese. Obesity impacted several behavioral parameters. First, in the holeboard discrimination task, obese animals showed better memory performance but took longer to start (first visit latency) and complete the test, expressed lower reward seeking behavior to spend more time exploring walls, increased vocalizations emission and reduced vigilance reactions toward environmental sporadic events (sudden noises). Obese animals ate less than lean animals during the food choice test, but significantly increased “bite” and “sniff” frequencies.

In the holeboard discrimination task, the higher WM scores observed in obese animals must be interpreted in the light of their poor motivation to seek for the food rewards: animals showed a loss of interest toward the food rewards, and reduced strongly the number of bowls’ explorations, resulting in decreased errors number. Furthermore, animals first experienced the test in Study 1 and a better understanding of the test by learning cannot be ruled out. As a consequence, the WM results must be interpreted with caution. Furthermore obesity is often associated with impaired cognitive functions^3,38^ rather than improved cognition.

Before the holeboard discrimination task, animals received only ¼ of their daily ration (*i.e.* 250 g of HFS feed matching with the allocated ration during the tests in Study 1), which means that animals were not sated before this test. Under these conditions, and knowing that animals ate their entire ration after the test (~ 700 g), we can assume that animals were hungry during the holeboard task, which dismisses satiety as an explanation for the loss of interest in seeking food rewards. Then, the higher test duration and slower shifting from one bowl to another when obese could not be explained by potential locomotor difficulties related to their weight status, for two reasons: first, obese animals reallocated significant time to wall exploration and not to resting state, and second, according to the work of Shin et al.^39^, diet-induced obese rats are less motivated to work for food rewards than chow-fed lean rats, suggesting a loss of *wanting* (*i.e*. motivation). Obese rats seem to respond only to easily accessible palatable stimuli, such as obese minipigs in the present study. They continued to eat during the two-choice test, although significantly less than in Study 1, despite of the fact that they received their total ration before the test. Interestingly, obese animals increased their “bite” frequency during the two-choice test. This behavioral pattern is also observed among obese people: obese or overweight preschoolers and adults ate at a faster rate, taking more bites and chewing each bite fewer times, and failed to show the normal pattern of slowing-down the eating rate toward the end of the meal compared to normal-weight people^40–42^.

Moreover, in addition to increased walls exploration suggesting a loss of interest toward the test (reward), as discussed above, obese animals increased the number of vocalizations. The vocalisations might reveal a search for social contacts. Similarly to what is known in many social species, being socially isolated constitutes a major stressor for pigs^43–45^. The fact that the need for social contact appeared stronger in obese than in lean animals is in line with current knowledge about the effects of obesity and/or high palatable food consumption on anxiety- and depression-like behaviors in human^2,46,47^ and animal models^48,49^. Obese animals showed higher frequencies of “sniff” occurences during the two-choice food test. Upstream of pathology, a chronic exposure to a Western-type diet impairs olfactory acuity^50,51^, as well as obesity^50,52^ but it was not observed in Study 1, where lean SD and WD groups expressed the same number of “sniff” behavior.

### Study 3: Weight loss intervention effect

After intervention, both RYGB and Sham animals experienced a significant weigt loss (-15.9% and -10.6% respectively), shifting from morbidly to moderately obese, which is determinant for decreasing the risk of comorbidities in the human^53^. Significant improvements were observed after weight loss in terms of behavior and brain responses to sucrose, and the obesity surgery had different effects compared to diet restriction only.

During the holeboard discrimination task, memory scores were similar between obese and post-intervention status. As previously discussed, memory scores of obese animals are not easily interpreted. In the literature, short- and long-term memory improvements have been shown after bariatric surgery^54–56^. Yet, dieting and a high level of dietary restraint have been associated with cognitive impairment^57,58^, which was not observed in the present study. As well as in Study 2, a learning and memory retention from previous study cannot be ruled out, and could explain better scores in Study 2 (obese) and Study 3 (operated/restricted animals) than in Study 1 (lean animals). Additional cognitive tests would be relevant to assess actual memory skills of the animals.

After both RYGB and Sham interventions, during the holeboard discrimination task, animals recovered a motivation level higher than in Study 2 and comparable to Study 1 according to their shorter test duration, vocalizations emissions, switch time, and wall exploration, and opposed to their higher number of visiting bowls, without any difference between RYGB and Sham groups. According to the literature, these results might reflect a better welfare permitted by the weight loss interventions. As a matter of fact, bariatric surgery appears to foster improvement of well-being in the majority of morbidly obese patients^59^. Moreover, after bariatric surgery, patients reported a decreased influence of emotions on eating behavior^60^, which is in line with our results showing a stress-less emotional status in animals. Conversely, contrasting results were highlighted for dieting practice (comparable to our sham intervention): dieters have higher salivary cortisol and perceived stress^61^, if not related to, at least associated with food frustration^62,63^.

In the two-choice tests, weight loss intervention resulted in higher food consumption (RYGB and Sham pooled together). More precisely, RYGB tended to increase their food intake while Sham significantly increased their food intake compared to the obese status in Study 2, and RYGB tended to eat less than Sham animals. A greater difference was expected between Sham and RYGB because of the small size of gastric pouch in RYGB compared to the normal entire stomach of Sham, but an important interindividual variability was observed within the RYGB group. In the literature, although RYGB intervention remains one of the best options to induce weight loss by decreasing food intake^64^, there is still a large proportion of patients who do not loose weight or quickly regain weight^65^, which is associated with increased food intake in the form of snacking^65–67^. This “snacking-type” behavior observed in our obese minipigs (higher “bite” frequency and food intake without hunger) persisted in both RYGB and Sham animals.

Higher brain activity was observed in P-SC, SAC (sensory processing areas), insula (gustative area), aPFC (decision making based on hedonic estimation) and fornix (memory) in response to oral sucrose stimulation in PET imaging, after weight loss intervention (RYGB and Sham pooled together). The fornix is the principal tract associated with hippocampal connections beyond the temporal lobe^68^, and a negative correlation between BMI and white matter in the fornix was shown^69,70^. Hippocampal-prefrontal cortical circuit mediates inhibitory response control in the rat^71^. The hippocampus is densely populated with both leptin and insulin receptors^72^, and in consequence, sensitive to satiety signals^73^. Thereby, brain memory pathway plays a crucial role in eating behavior, and a higher response in the fornix could reveal a better communication within memory-sensory and cognitive areas. When it comes to the aPFC, this area (BA10) can be subdivided into three different subareas (10p, 10 m, 10r) and include the ventromedial PFC (vmPFC, 10 m)^74^. The vmPFC has already been shown to act in synergy with the OFC in expected value computation, reward outcome and experienced pleasure. It implements categorical decision processes, transforming the value signals into a choice consequently orienting behavior^75^. To summarize, vmPFC (BA10) seems to be involved in choices based on reward value previously evaluated by the OFC. In the context of our study, a higher activation of the aPFC in response to sweet taste might be related to a better modulation of the positive outcomes driven by sweet perception and therefore might lead to a better control of eating behavior. Caloric restriction also led to neuronal reworking in humans, involving higher responses in insula (short-term effect), caudate nucleus, and hippocampus^76^.

RYGB and Sham differed in brain responses to sucrose: RYGB showed higher responses in the dorsal striatum (Cau, Put) and PP. The dorsal striatum plays a major role in stimulus-reward association. The Cau has been shown to be involved in working memory thanks to its connection with Hpc and amygdala^77,78^, but it especially interferes with the inhibitory control of action^79,80^. Functional impairment of Cau has been linked to higher impulsivity^81^ of eating behavior. The caudate activity modulates the Put, involved in habits, *i.e.* usual behaviors^81,82^. The synergetic action of both structures manages motor planification, and a higher response in the Cau could be related to higher inhibitory control. The prepyriform area is part of the olfactory area^83^, and lesion of this region resulted in increased food intake^84^ in rats. These data might confirm the importance of olfaction in eating behavior and match some of our own results, except for olfactory sensitivity that remained altered according to our behavioral data.

## Conclusion and perspectives

This work contributed to highlight the segregated effects of Western diet consumption and obesity, as well as the differencial outcomes of the gastric bypass compared to a restrictive diet, in terms of behavior and brain metabolism. Chronic exposure to WD in lean adolescent minipigs led to “addict-like” brain activation patterns, but without behavioral issues likely to favor risky behavior and eating habits under challenging environments (*i.e.* sensitivity programming). Obesity altered eating behavior inducing a snacking-type meal associated with an increasing “sniff” occurencies in line with a loss of olfactory acuity described in the literature. Obesity also increased anxiety-like behaviors. Weight loss interventions (RYGB and restriction) partially restablished normal behavior, and enhanced brain responses in the striatum and prefrontal-hippocampal pathway.

## Materials and methods

The experiments presented in this paper were conducted at INRAE St Gilles (Agreement No 3527532), France, in accordance with the current ethical standards of the European Community (Directive 2010/63/EU). This protocol was approved by the Ile-et-Vilaine Ethics Committee No 007 (agreement No 201504280924565) and the Ministry of Higher Education and Research (Reference No APAFIS #598–201504280924565 v5).

### Housing conditions

The animals were kept in a room of 50 m^2^, maintained at a temperature of 22 °C and equipped with artificial lighting in L / D 15:9. As soon as they entered the experiment, the animals were housed in individual pens measuring 110 × 80 cm of surface and 110 cm high. The bars spaced 8 cm apart allowed contact between animals in adjacent pens. These cages were equipped with individual 50-cm-long troughs. Each animal had access to water ad libitum, and had a metal chain for play. The animals remained in these accommodation conditions throughout the duration of the protocol. Body weight and food refusal were measured every week throughout the protocol.

*Study 1: Normal-weight & Isocaloric. The goal of Study 1 was to investigate the effects of a short-term chronic exposure to a Western-type diet, without weight gain, compared to a healthy standard diet.* For this study, 20 animals were exposed to high-fat-sucrose (HFS) diet (defined as the Western diet WD group), and 8 animals received a standard (STD) diet. SD and WD animals were kept at a normal weight during the first stage of the experiment, which explains why WD animals from Study 1 are called lean WD. Exposure to the WD began 8 weeks prior to initiation of behavioral testing. The WD provided 10.8 MJ/kg of feed and the SD 7.27 MJ/kg of feed, expressed in net energy (NE). The nutritional requirements of a minipig are calculated on the basis of the metabolic weight (MW = LiveWeight^0.75^ for the basal metabolism). The dietary intake was adjusted continuously according to the evolution of the body weight. The composition of both diets is described in Table 2.

**Table 2:** Composition and nutritional values of the feeds used along the experiment.

Composition (%)	Standard (STD)	High-fat sucrose (HFS)
**Animal's diets**		
Wheat	10	6.25
Barley	33	12
Wheat bran	25	14
Soybean meal	6	12
Sunflower meal	10	8
Soybean hulls	12	8
Molasses	1	
Corn starch		6.5
Sucrose		20
Lard oil		10
Bicalcium phosphate	0.6	0.6
Calcium carbonate	1.3	1.3
NaCl	0.6	0.6
Mineral vitaminic complement	0.5	0.75
Total	100	100
Metabolisable energy (MJ/kg)	10.31	14.09
Net energy (MJ/kg)	7.27	10.80
**Nutritional value (%)**		
Dry matter	87.7	91.8
Cellulose	11.05	7.49
Glucids (starch)	28.23	38.34
Lipids	2.17	11.38
Nitrogen matter	15.22	12.74
Mineral content	6.81	5.97

*Study 2: Obesity development. The goal of Study 2 was to investigate the effects of weight gain induced by a long-term chronic exposure of animals to a Western-type diet when compared to their previous lean status in Study 1.* This phase involved the 20 WD animals used in Study 1. The WD animals were exposed to the obesogenic diet, which corresponded to ad libitum access to the HFS feed. Individual consumption was measured throughout the entire period. The animals were rationed from the 15th week of obesogenic diet (W15, when the 60% excess weight was reached, see below) according to a gradual restriction plan, to limit weight gain. Because no consensus has been reached to define obesity in most animal models, obesity was defined in this paper as a > 20% weight gain compared to the maximum theoric healthy weight. As a matter of fact, according to human BMI scale, overweight is characterized by a ]0:20] % weight gain compared to the maximal normal healthy weight, moderate obesity is reached when subject present ]20:40] % of excess weight, severe obesity for ]40:60] % and morbid obesity for > 60% weight gain from healthy weight. Because animals were still growing during the experimentation, initial healthy weight record in one-year-old animals could not be used as a reference to calculate suitable percentage of weight gain during the obesogenic challenge, which ended when the animals were 2 years old (weight stabilization in Yucatan minipig is reached around 3 years old). Thus, the weight status was determined on the basis of actual weight compared to the Yucatan minipig theory healthy weight curve (personal communication, provided by UEPR, INRAE, Saint-Gilles, France).

*Study 3: Weight loss interventions. The goal of the Study 3 was to investigate the effects of weight loss induced by either by a gastric bypass or a caloric restriction when compared to previous obese status in Study 2.* This phase was designed to involve 16 of the 20 obese animals following the obesogenic phase in Study 2. The 4 remaining unselected animals involved 2 individuals that died during the experiment (one during the obese phase due to cardio-respiratory issues, and the other due to post-surgery complications), 1 animal presenting motivational issues during behavioral testing from Experiment 2, and the last one did not undergo PET-scan imaging in Experiment 2. Weight loss was induced using two different strategies: a group of 8 animals underwent a Roux-en-Y gastric bypass (RYGB group) while 8 animals were subjected to a restrictive diet, in a pair-feeding plan calculated according to the feed consumption recorded in the RYGB group; in order to avoid the confounding effect of surgery (*i.e.* anesthesia, postoperative recovery, antibiotic and analgesic treatment, stress), the restricted group was sham-operated (Sham group).

*Roux-en-Y gastric by-pass bariatric surgery.* Pre-anesthesia was performed by intramuscular injection of ketamine (5 mg/kg – Imalgene 1000, Merial, Lyon, France), followed by isoflurane inhalation (Aerane 100 ml, Baxter SAS, France) used to induce the suppression of the pharyngotracheal reflex and then a surgical level of anesthesia, 3–5% v/v and 2–3% v/v respectively. Respiratory frequency was adjusted at 15 breathing/minute, tidal volume between 420–470 ml and ETCO2 maintained between 3.8 and 4.5%. The management of pain was ensured by morphine (fentanyl citrate) intravenous injection with a 0.4 m L/minute debit for a total of 10 mL administration (Fentanyl Renaudin 50 µg/mL, lab. Renaudin – 64,250 Itxassou, France), and hydric homeostasy was ensured thanks to a Ringer’s lactate perfusion with a debit of 22 mL/min (Ringer Latate, B. Braun Medical, Virbac France – 06,517 Carros, France). The surgical procedure was performed by abdominal laparotomy, and involved the creation of a 30-mL gastric pouch, connected to a 150-cm Roux-limb, this one being also connected to a 70-cm billio-pancreatic limb, as described in Verhaeghe et al.^85^.

*Post-operative care*. After surgery, pain management was ensured by morphine sub-cutaneous injection at 0.5 mg/kg (Morphine Chlorhydrate Renaudin, lab. Renaudin – 64,250 Itxassou, France). Antibioprophylaxis was performed the day of surgery and two days later using intramuscular injection at 15 mg/kg each time (Dufamox LA, Zeotis, 75,014 Paris, France).

## Behavioral testing

### Spatial holeboard discrimination task

*Test area and apparatus.* The testing procedure is the same as described in Gautier et al. ^35^, except that test duration was reduced here to 5 min and only two entry doors were used instead of four.

*Data recording and analysis.* Virtual square areas (≈1m^2^ each), numbered from 1 to 16, corresponded to each bowl and were used as localization items; the behavior of the animals was recorded in order to assess motivation for test/reward (test duration, first visit latency, reward ratio as the number of eaten reward divided by the number of available rewards, switch time, number of bowls’ visits), emotional/attentional status (immobility, exploration of the walls, vigilence and vocalization), and memory (working memory WM and reference memory RM) as:

$${\text{WM}} = \frac{{{\text{Number}}\;{\text{of}}\;{\text{baited}}\;{\text{visits}} }}{{{\text{All}}\;{\text{visits}}\;{\text{to}}\;{\text{the}}\;{\text{baited }}\;{\text{bowls}}}}\quad {\text{RM}} = \frac{{ {\text{Number}}\;{\text{of}}\;{\text{visits}}\;{\text{to}}\;{\text{all}}\;{\text{bowls}}}}{{{\text{Total}}\;{\text{number}}\;{\text{of}}\;{\text{visits}}\;{\text{to}}\;{\text{all}}\;{\text{bowlts}}}}$$

Behaviors were recorded in real time during the three phases of the holeboard test under the Observer Noldus software (Noldus Information Technology, B.V., 2013. The Observer XT Base Package, Version 11.5, Wageningen, The Netherlands). Behavioural sampling was done according to the ad libitum continuous focus sampling method.

### Two-choice feed test

This feed choice test was carried out on animals having received their entire daily ration the morning before the tests. Two different feeds were presented simultaneously in a free-choice situation directly in animals’ familiar pen: a High Sucrose (HS) feed, consisting of a coarse crushed standard minipig feed containing 20% ​​sucrose, and an identical but Low Sucrose (LS) feed containing 2% sucrose.

One of the bowls contained 500 g of HS, while the other bowl contained 500 g of LS; the two feeds being mixed in 150 ml of water in order to modify the feed texture compared to the usual granulated feed. This choice was offered to the animals once a day for four days, in the morning, alternating each day the position of the feed in the right bowl and the left bowl in order to avoid any laterality bias. Animals had access to feed for up to 10 min. The test stopped either when the animal finished one of the feeds or after 10 min elapsed. For each test the refusals (type(s) of feed(s) and quantity), the amounts of feed and calories consumed, the first feed chosen, *i.e.* eaten, and the latency of first consumption, occurrences and durations of “eat” and “smell” behaviors for each HS and LS feeds were measured. Ingestion rate (g/min) and behaviors frequencies (N/min) were calculated.

### Positron emission tomography imaging (PET)

*Principle*. The goal of this imaging modality was to map the brain glucose metabolism, in response to oral sucrose stimulation, by injecting a radioligand, 18F-FDG (2-fluoro-2-deoxy-D-glucose—FLUCIS 2173 MBq/ml).

*Animal anesthesia and radioligand administration*. The imaging cohort consisted of 24 animals in Study 1 (16 WD and 8 SD), and 16 animals in Study 2 and 3, with a sex ratio of 50/50 in each group. The animals were fasted (food only, water still ad libitum) the day before imaging to maintain blood glucose lower than 6 mmol/L on the day of imaging. The modalities of anesthesia and radioligand injection are fully described in Gautier et al. (2018). The 18FDG injection was performed when the animal had a stabilized MAC (Minimum Alveolar Concentration) at: (i) 2.0 in normal-weight (Study 1), (ii) 1.5 in Obese (Study 2) and Weight loss intervention (Study 3) phases. The animals were in the prone position (Head First Prone) in Study 1, and shifted in supine position (Head First Supine) in Study 2 & 3, because of anaesthesia issue related to cardio-respiratory difficulties in obese animals (the prone position induced more organ compression, which was difficult to cope with in overweight anaesthesied animals).

*PET imaging with oral sucrose stimulation protocol*. PET imaging was performed with a Siemens HR + (Siemens Ecat, 962, HR +). Scout scan was performed 30 min prior to injection to check the correct positioning of the animal. A transmission tomography (Ge 68) was performed before the injection to calculate the signal attenuation. Injection of the radioligand was performed at the time of oral stimulation. The acquisition started 45 min after the radioligand injection and lasted 40 min. Finally, the images were reconstructed in a 6-iteration process (16 subsets, zoom = 8) using a 6-mm Kernel FWHM to obtain a continuum of 63 sections with spatial resolution 0.64 mm per pixel on the x and y axes and 2.42 mm on the z axis. The stimulation device consisted of a computer-assisted automate (gustautomate) controlling the parameters and synchronization of the diffusion of the solutions tested on the tongue of the animal via a lingual catheter. The vehicle solution was an artificial saliva solution of the following composition: NaCl (4 mM), KCl (10 mM), NaHCO3 (6 mM), KHCO3 (6 mM), HCl mM), MgCl2 (0.5 mM), K2HPO4 (0.24 mM), KH2PO4 (0.24 mM). The sucrose solution consisted of artificial saliva (vehicle) containing 16% sucrose (Fisher Chemical, Bishop Meadow Riad, Loughborough, Leic., LE 11 5 RG, UK). The oral stimulation protocol consisted of a 5-min rinse with artificial saliva at a flow rate of 24 ml/min, then animals were stimulated with sucrose, concomitant with the injection of the radioligand, for 15 min at a flow rate of 24 ml/minute. Finally, a second artificial saliva rinse of 15 min at 24 ml/min was performed.

*Images preprocessing*. The process was the same as described in Gautier et al., 2018. Raw brain uptake value was adjusted using standard uptake value (SUV) normalization based on lean body composition following the formula:

$$SUV = \frac{{{\text{Raw}}\;{\text{uptake }} \times \left( {{\text{Weight}}\;\left( {1 - {\text{\% Fat}}} \right)} \right)}}{Radioactivity\; injected \;dose}$$

With weight (kg), raw uptake (MBq/cm^3^), radioactivity injected dose (MBq).

### Statistical analysis

Statistical analysis was performed using R software (R Development Core Team (2005). R: A language and environment for statistical computing. R Foundation for Statistical Computing, Vienna, Austria. ISBN 3–900,051-07–0, URL: https://www.R-project.org) for all behavioral analysis.

*Weight* was analyzed using t-test to compare lean SD *vs.* lean WD, and RYGB *vs.* Sham, and paired t-test to compare Obese WD *vs.* lean WD, and Obese WD *vs.* Intervention (RYGB and Sham pooled together and separated).

*Behavioral data* were analyzed with a non-parametric approach, using Wilcoxon signed rank test for paired samples: Obese *vs.* lean WD, and Obese *vs.* Intervention, Intervention (RYGB and Sham pooled together and separated), and Wilcoxon-Mann–Whitney U-test for independent samples (lean SD *vs.* lean WD, and RYGB *vs.* Sham). The quantities of HS and LS feed intake during the two-choice tests were compared using Wilcoxon signed rank test for paired samples (HS *vs.* LS).

*Brain PET imaging* was analyzed under SPM 12 with three different contrasts: lean WD *vs*. lean SD, Weight loss (Study 3) *vs*. Obese WD (Study 2), and RYGB *vs*. Sham. Whole-brain analyses were followed by ROIs analyses on the following bilateral brain areas: dorsolateral prefrontal cortex (dlPFC), anterior prefrontal cortex (aPFC), orbitofrontal cortex (OFC), caudate nucleus (Cau), putamen (Put), nucleus accumbens (NAc), globus pallidus (GP), insular cortex (Ins), hippocampus (Hpc), parahippocampal cortex (PHC), somatosensory association cortex (SAC), ventral posterior cingulate cortex (vpCC), ventral anterior cingulate cortex (vACC), dorsal posterior cingulate cortex (dpCC), and dorsal anterior cingulate cortex (dACC). These brain areas were selected upon a priori hypotheses, to investigate neural networks specifically involved in food reward and cognitive processes, attentional and executive functions. Family-wise error (FWE) correction was systematically applied (with *P* < 0.05 after correction), but uncorrected significant differences at *P* < 0.001 were also indicated since for some contrasts (*e.g.* for the lean WD *vs*. lean SD and RYGB *vs*. Sham contrasts), no differences were observed after FWE correction.

## Data Availability

Upon acceptance of the paper, raw data will be provided via a public repository platform such as Dryad.

## Abreviations

aPFC: anterior prefrontal cortex
BA: Brodmann areas
Cau: caudate nucleus
dACC: dorsal anterior cingulate cortex
dlPFC: dorsolateral prefrontal cortex
dpCC: dorsal posterior cingulate cortex
Fx: fornix
GP: globus pallidus
HFS: high-fat-sucrose feed
Hpc: hippocampus
HS: high sucrose
Interv: Intervention (RYGB and Sham pooled together)
Ins: insula
LS: low sucrose
Nac: nucleus accumbens
OFC: orbitofrontal cortex
P-SC: primary somatosenory cortex
PET: positronic emission tomography
PHC: parahippocampal cortex
PP: prepyriform are
Put: putamen
RM: reference memory
ROI: region of interest
RYGB: group subjected to a Roux-en-Y gastric bypass
SAC: somatosensory association cortex
SD: standard diet group (fed with STD feed)
Sham: sham-operated group
STD: standard feed
SUV: standardized uptake value
SVC: small volume correction
vACC: ventral anterior cingulate cortex
vmPFC: ventro-medial PFC
vpCC: ventral posterior cingulate cortex
WD: western diet group (fed with HFS feed)
WM: working memory
